# SMART Drumlines Ineffective in Catching White Sharks in the High Energy Capes Region of Western Australia: Acoustic Detections Confirm That Sharks Are Not Always Amenable to Capture

**DOI:** 10.3390/biology11101537

**Published:** 2022-10-20

**Authors:** Stephen M. Taylor, Jason How, Michael J. Travers, Stephen J. Newman, Silas Mountford, Daniela Waltrick, Christopher E. Dowling, Ainslie Denham, Daniel J. Gaughan

**Affiliations:** Western Australian Fisheries and Research Laboratories, Department of Primary Industries and Regional Development, Government of Western Australia, P.O. Box 20, North Beach, WA 6920, Australia

**Keywords:** bather protection, shark attack, human–wildlife conflict, power analysis

## Abstract

**Simple Summary:**

Shark hazard mitigation measures are often introduced after human-shark interactions, which are increasing. Such measures are often contentious, and care is needed to reduce the risk to ocean users without causing negative ecosystem impacts. Here, we examined the effectiveness of Shark-Management-Alert-in-Real-Time (SMART) drumlines to catch, tag, relocate and release white sharks (*Carcharodon carcharias*) in the Capes region of Western Australia. The project aimed to examine the movement patterns of white sharks’ post-release to determine whether their relocation provided a reduction in risk for ocean users. The effectiveness of the program was also evaluated in terms of minimising the mortality of all animals caught. In total, 352 fish were caught over the 27-month trial period and 91% of animals were released alive in good condition. Only two white sharks (target species) were caught, both of which moved immediately offshore after capture and remained predominantly in offshore waters for the duration of tag deployment. The detection of 24 other tagged white sharks within the acoustic array during the trial period confirms that the target species were not always amenable to capture. Our results reiterate there is no simple remedy for dealing with the complexities of shark hazards and reinforce the importance of trialing mitigation measures under local conditions.

**Abstract:**

The management of human-shark interactions can benefit from the implementation of effective shark hazard mitigation measures. A Shark-Management-Alert-in-Real-Time (SMART) drumline trial in the Capes region of Western Australia was instigated after several serious incidents involving surfers and white sharks (*Carcharodon carcharias*). The project aimed to determine whether white sharks (target species), which were relocated after capture, remained offshore using satellite and acoustic tagging. Over a 27-month period, 352 fish were caught, 55% of which comprised tiger sharks (*Galeocerdo cuvier*). Ninety-one percent of animals were released alive in good condition. Only two white sharks were caught; both were relocated ≥ 1 km offshore before release and moved immediately further offshore after capture, remaining predominately in offshore waters for the duration of their 54-day and 186-day tag deployments. Our results confirm that desirable animal welfare outcomes can be achieved using SMART drumlines when response times are minimised. The low target catches and the detection of 24 other tagged white sharks within the study area supported the decision to cease the trial. Our results reiterate there is no simple remedy for dealing with the complexities of shark hazards and reinforce the importance of trialing mitigation measures under local conditions.

## 1. Introduction

Human-shark interactions are often traumatic events that cause negative social and economic impacts on affected communities [1]. Such incidents are rare, with a confirmed 73 unprovoked shark bites on humans worldwide in 2021 (https://www.floridamuseum.ufl.edu/shark-attacks/yearly-worldwide-summary/ Accessed on 1 July 2022); however, the number of reported shark bite incidents and fatalities has risen since records began [2], causing increasing levels of community concern [3]. The negative impacts of these incidents has led to a variety of shark hazard mitigation measures being implemented that are predominantly designed to reduce the likelihood of ocean users being bitten [4,5,6,7]. Historically, most shark hazard mitigation measures were designed to kill target species, with less emphasis on minimising impacts on harmless species [8,9]. The longest on-going shark hazard mitigation measures involve the use of large-mesh gillnets and drumlines to target larger sharks off beaches in KwaZulu-Natal, South Africa [10], New South Wales [11] and Queensland, Australia [12]. Modifications to gear design and deployment has occurred in all three of these programs to reduce the impact on non-target species, although varying levels of mortality continue to occur from these incidental catches [12].

There is growing public support for shark mitigation measures to have minimal environmental impact and mounting pressure to consider other approaches to reduce mortalities [10,13,14,15]. Shark-Management-Alert-in-Real-Time (SMART) drumlines are intended to be non-lethal [16]. The capture of an animal on a hook is relayed to the user in real-time, based on an adapted trigger mechanism linked to a GPS buoy and connected to the Iridium satellite. This assists in minimizing the time animals spend on the hook, thereby improving the welfare of captured species. This mitigation measure was first used at Réunion Island in the Indian Ocean to target bull (*Carcharhinus leucas*) and tiger (*Galeocerdo cuvier*) sharks [16,17]. The approach has also been used in New South Wales (NSW), Australia to target white (*Carcharodon carcharias*), bull and tigers sharks [18,19] whereby sharks are caught and relocated 1 km offshore. The use of SMART drumlines has resulted in fewer animal mortalities in comparison with traditional drumlines [12,16,19,20]. While these SMART drumline and relocation studies have provided promising results in terms of reducing the short-term risk to ocean users and minimising bycatch, two separate white shark populations occur in the different marine systems off the western and eastern coast of Australia [21,22]. This could lead to regional differences in the behavioural responses to capture and overall performance of SMART drumlines.

**Figure 1:**
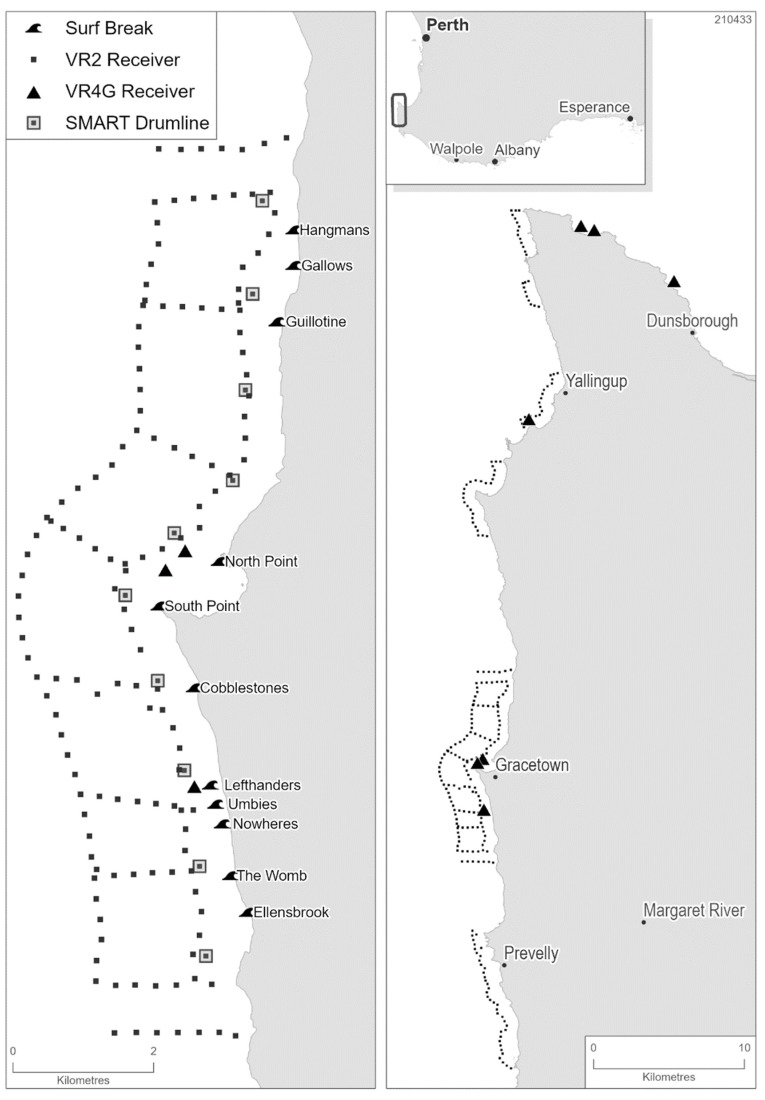
Map of the Capes region of Western Australia highlighting the 10 fixed locations where SMART drumlines were deployed and the locations for the VR2 and VR4G or Rx LIVE receivers. Left panel displays the location of the drumlines in relation to popular surf breaks and the receivers within the Gracetown array. Right panel displays the primary and secondary receiver arrays and the location of the Capes region in relation to the state capital of Perth.

In Western Australia (WA), there has been an increasing trend in shark bite incidents since the 1970s [2,23]. Fifteen fatalities occurred between 2000 and 2017, 11 of which occurred between 2010 and 2017. All fatal shark bite incidents that occurred over this latter period reportedly involved white sharks. In response, a range of studies led by the Department of Primary Industries and Regional Development (DPIRD, Government of Western Australia) have improved the understanding of the movement ecology of white sharks [24,25], the potential impacts of fishing on population size [26,27,28], and potential risk factors associated with white shark bite incidents in WA [29]. Following several serious surfer-shark incidents in the Capes region in 2018, the use of SMART drumlines was suggested as a mitigation measure by members of the community. Thus, the aim of this study was to evaluate the efficacy of SMART drumlines in reducing the level of risk to ocean users in the Capes region (Figure 1), to assist in determining whether this measure could be integrated into the suite of existing shark hazard mitigation strategies in WA (https://www.sharksmart.com.au/ Accessed on 5 December 2021). This involved examination of the (i) response times, hooked times and release condition for all target and non-target species; (ii) movement patterns of white sharks caught on drumlines and relocated offshore; (iii) and the detection of other white sharks throughout the study area that were not caught on drumlines.

## 2. Materials and Methods

### 2.1. Power Analysis

An a priori power analysis was undertaken to investigate the relationship between sample size (i.e., number of caught, relocated, and released white sharks) and the associated power of the experiment to detect a difference in the proportion of success (i.e., the proportion of white sharks not returning, resulting in risk reduction). The analysis compared the effect of the mitigation measures on the proportion of success ($p_{1}$) to the base level (i.e., $p_{0}$ for no mitigation measure). A small, medium, or large effect size (ES) was deemed to correspond to an increase in proportion of approximately 0.09, 0.22, or 0.34, respectively, from the base level, following the arcsine transformation of the proportions ($ES=2\mathrm{asin}\sqrt{p_{1}}-2\mathrm{asin}\sqrt{p_{0}}$) and considering base proportions from 0.2 to 0.8. This broad range in base proportions reflected the uncertainty in knowledge of white shark movements in the study region.

### 2.2. SMART Drumlines

#### 2.2.1. Consultation and Design of Trial

The scientific framework for the trial was decided following community consultation, including the configuration of the SMART drumlines in the Gracetown area. A SMART Drumline Trial Ministerial Reference Group (Reference Group) was formed, with representatives from State and Local Government Agencies, the Conservation Council of Western Australia, Sea Shepherd, Surfing Western Australia, and Surf Life Saving Western Australia. The Reference Group assisted in many aspects of the trial, provided regular feedback on the process, and assisted in communicating the trial objectives and results to interested community members. The configuration of the SMART drumline locations surrounding Gracetown was open to public consultation from 13 September 2018 until 10 October 2018. The preferred option was that 10 SMART drumlines be deployed evenly (a similar distance apart), about 500 m from shore, along an 11.5 km of coast that contained at least 11 surf breaks (Figure 1).

#### 2.2.2. Daily Operations, Data Collection and Analysis

Weather permitting, 10 drumlines were deployed and retrieved daily by a commercial contractor to DPIRD. Prior to commencement, all crew were trained in animal handling techniques. Oversight of fishing operations and animal handling was facilitated by DPIRD observers, and an observer was on-board for 30.8% of all fishing days. Additionally, independent third-party observers representing the Conservation Council of Western Australia and Sea Shepherd were on-board the vessel for 1.7% of all fishing days.

Commencement of SMART drumline deployment occurred no later than one hour after sunrise and was completed no later than two and a half hours after sunrise. The delayed commencement of fishing operations was approved for periods involving risk weather conditions or when there was congestion at the boat ramp. Retrieval of SMART drumlines did not occur earlier than two hours before sunset and was completed by sunset. If weather conditions deteriorated and the safe handling of animals or safety of personnel was compromised, fishing gear was retrieved, and operations ceased for that day.

The overall deployment of SMART drumlines was broadly consistent with their use in NSW. Each SMART drumline was attached to a single Mustad Giant Circle Hook 20/0 (39937NP-DT) that was baited with either Western Australian salmon (*Arripis truttaceus*) or sea mullet (*Mugil cephalus*) approximately 1 kg in weight. The bait was stored and transported frozen, then defrosted within 24 h of being used during fishing operations. Each bait was suspended ~2–2.5 m below the surface during calm conditions. However, during periods of strong surface currents, the bait could be pushed closer to the surface. The depth at each drumline location ranged between 11–30 m. Baits were intentionally set in the upper water column because the white shark is a pelagic species and to mitigate against the bycatch of demersal finfish and elasmobranch species. Drumlines were checked every three hours and empty hooks or those where part of the bait had been removed were re-baited. These regular checks were also designed to minimise harm to any hooked animals that may not have triggered the alarm. In the event of an alarm (i.e., trigger activated on the GPS buoy), the fisher was required to attend the triggered SMART drumlines within 30 min, and to determine whether an animal was on the hook or if it was a false alarm.

Upon capture, sharks were secured to the vessel in accordance with DPIRD tagging procedures such that pain and distress were minimised (e.g., shark’s head and gills were submerged at all times), aligned with standard operating procedures developed for larger sharks [30]. Once secured, each animal was identified to species and length measures were taken and the sex of each shark was recorded. A yellow identification tag (Hallprint, PDAT dart tag) was inserted in all animals at the base of the dorsal fin. White sharks were also fitted with an external acoustic tag (Innovasea, Boston, MA, USA, V16-6H) and pop-up satellite archival transmitting tag (PAT; Wildlife Computers, miniPAT 348). Once all data collection and tagging was completed, the animal was released. All white sharks and tiger sharks ≥ 3 m in total length (TL) were relocated at least 1 km offshore when both the health and safety of the crew and shark could be maintained. If adverse weather conditions occurred at the time of capture, these sharks were released without relocation.

The response time was defined as the boat arrival time minus the activation time (in minutes). The hooked time was defined as the animal release time minus the activation time (in minutes). Where possible, the hooking location was categorised as: Corner (hook in corner of the jaws); Mouth (hook inside the mouth); Swallowed (hook likely not visible) and Foul hooked (hook outside of the mouth or jaws). On those occasions when the hook could not be removed, the trace was cut as close to the hook as possible. Release condition was initially assessed visually by the crew upon release and subsequently validated by examining underwater footage of the release of each animal, captured from a pole-mounted GoPro camera. The release of each animal was assigned: 1 = Animal swam away strongly in good health; 2 = Swam away slowly; 3 = Failed to swim away and sunk, chances of survival appear low; 4 = Animal died; 5 = Animal euthanised because of injuries.

The catch of each species was combined from all 10 SMART drumline locations and the hypothesis that the sex ratio of sharks was 1:1 was tested with the χ^2^ statistic. A significance level of *p* < 0·05 was required for rejection of the null hypothesis [31]. This statistical analysis was restricted to only those species where >20 animals were caught.

### 2.3. Assessing Fine-Scale Movement

Acoustic receivers (*n* = 240; VR2W, Tx, AR; Innovasea) were deployed in six arrays in the Capes region and were complimented by five near real-time acoustic receivers (VR4G or Rx LIVE). The primary array of acoustic receivers was located off Gracetown and encompassed the 10 SMART drumlines (Figure 1). The secondary arrays were located approximately 1 km offshore at other known surfing locations within the vicinity (Figure 1). Spacing of receivers in these arrays (herein referred to as “the Gracetown array”) was based on the results of range testing, such that detection ranges from adjacent receivers should overlap.

The Gracetown array was designed to determine the initial movements of relocated white sharks. The array consisted of an inshore line of receivers approximately 500 m from the shore (Figure 1). An associated offshore line complimented the inshore line and was located approximately 2 km from shore. Ten cross-shore lines joined the offshore and inshore lines creating a gated design [32]. Once a shark was captured on a SMART drumline and relocated 1 km directly offshore, its release would be between the inshore and offshore lines with “gates” to the north and south of the release location. Therefore, the direction of post-release movement in any direction could be established. The secondary arrays (Figure 1) were designed to detect if a relocated shark moved inshore to an adjacent surf break. They consisted of a single line of receivers approximately 1 km from shore, with receivers closer to shore at each end of the array to “box” out the area and permit detection of a white shark in the area. Both the primary and secondary arrays as well as the associated VR4 receiver at Meelup (adjacent to Dunsborough, Figure 1) permitted the detection of other acoustically tagged species.

To determine white shark movements within the arrays, fieldwork was scheduled at 12 and 24 months into the trial to retrieve, download/service, and re-deploy the VR2 receivers. As such, detections are reported for the first 24-month of the trial period, excluding the final 3-months when fishing still occurred. Movement data were reported as the number of white sharks detected within the Gracetown array and the number of “shark movement” events. Separate shark movement events were recorded when detections occurred more than 48 h since the last recorded detection for individual sharks on receivers in the Capes region.

### 2.4. Assessing Broad-Scale Movement

Each white shark caught in the trial was fitted with a PAT tag that was programmed to collect ambient light levels, temperature and depth at 300-second or 450-second sampling intervals, with data pooled into 12-h bins for histogram transmission. PAT tags were programmed to release after periods of 54 and 186 days in order to transmit their summarised data through the Argos constellation of satellites (www.argos-system.org Accessed on 1 March 2019) and enable the movement history to be estimated. Daily geographical positions were estimated using Global Position Estimator (GPE3) software, which runs within the Wildlife Computers’ Data Portal. The GPE3 software uses a Hidden Markov state-space model (time series) at a 0.25° grid resolution incorporating environmental variables, such as temperature, twilight observations, the diving depth of the animal, barriers to movement, and the maximum swimming speed of the study species, which in this study was estimated at 3.6 km h^−1^ [33].

## 3. Results

### 3.1. Power Analysis

**Figure 2:**
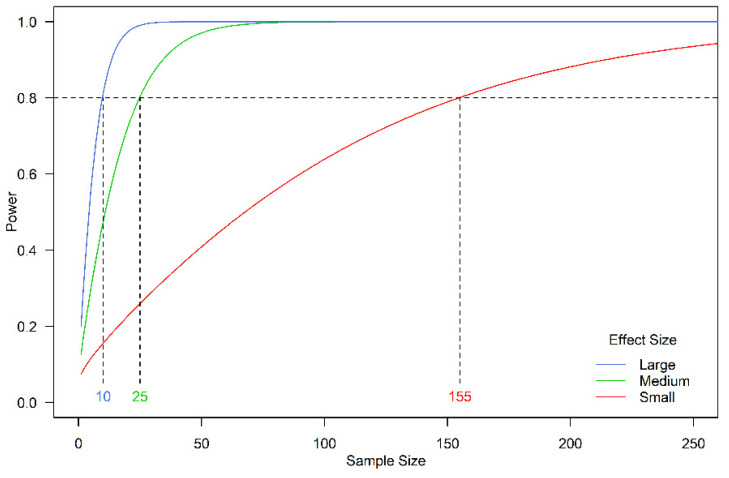
Relationship between the power of an experiment for varying sample size. This assumes a one-way test of one-sample proportion, with small, medium and large effect sizes specified by 0.2, 0.5 and 0.8, respectively, corresponding to an increase in proportion (from the base level with no mitigation) of approximately 0.09, 0.22 and 0.34 respectively. The effect size for two proportions are calculated using the arcsine transformation of the proportions. Numbers in colour denote the number of white sharks that would be required to be caught and relocated in order to detect an effect size.

Sample sizes of 10, 25 and 155 white sharks were required to statistically determine whether the capture, relocation and release of white sharks was effective in reducing the risk to ocean users (Figure 2). These numbers related to the sample size required to detect a large, medium or small positive effect size (i.e., reduction in risk to ocean users), respectively, with a minimum of 80% power, assuming that the effect actually exists. These sample sizes were all larger than the actual white shark catch (see Section 3.3).

### 3.2. Fishing Days and Response Times

Drumlines were deployed on 539 days between 21 February 2019 and 20 May 2021 (65.7% of all days). The drumlines were activated 937 times, of which 606 (64.7%) were ‘false alarms’ whereby there was no animal present on the hook upon checking the gear. The mean response time when an animal was hooked was 10.4 min (95% CI 9.8–11.0). On two occasions the 30-minute limit was exceeded (36 and 46 mins). In the first instance, the crew were dealing with a shark that had already been caught on another drumline, while in the other instance the crew were performing a bait check at the opposite end of the trial area.

### 3.3. Catch, Size and Sex Composition

In total, 352 fish were caught including two white sharks (target species; Table 1). Eleven fish had previously been caught during this drumline trial, resulting in 341 unique captures. Recaptured animals from the trial comprised 4 tiger sharks, 3 smooth stingrays (*Dasyatis brevicaudata*), 2 shortfin mako sharks (*Isurus oxyrinchus*), and 2 bronze whaler sharks (*Carcharhinus brachyurus*). Sharks numerically dominated (87.5%) the catch, while one stingray species and two finfish species contributed 10.5% and 1.9% to the catch, respectively. Of the seven species of shark recorded, tiger sharks comprised 54.5% of the overall catch and bronze whaler and shortfin mako sharks comprised a further 15.1% and 12.8% of the catch, respectively (Table 1). SMART drumlines captured sharks ranging in size from a 91 cm TL dusky shark to a 460 cm TL white shark. Sex ratios differed significantly from parity for tiger sharks and bronze whaler sharks, but not for shortfin makos (Table 1). Females were more commonly caught for tiger sharks, while males were more commonly caught for bronze whaler sharks.

### 3.4. Hooked Location, Hooked Time and Release Condition

In total, 71.5% of fish (including recaptures) were hooked in the corner of the mouth, 11.0% swallowed the hook, 9.0% were foul-hooked and 8.4% were hooked in the mouth. These percentages exclude those occasions when the fish “spat the hook” before the crew could confirm the hooking location. Foul-hooked animals were predominantly (77.4%) smooth stingrays and the majority of swallowed hooking events (86.8%) occurred for tiger sharks. The mean hooked time was 27.8 minutes (95% CI 26.7–28.9) and ranged from 5 to 73 minutes. A high percentage of animals (90.6%, *n* = 319) were released in good condition (release condition 1). In total, 7.7% (*n* = 27) of animals swam away slowly (release condition 2) and 0.6% (*n* = 2) failed to swim away and sunk (release condition 3). Three pink snapper were dead upon retrieval of the hook (0.9%, release condition 4) and an additional pink snapper was euthanized (0.3% release condition 5) due to barotrauma.

### 3.5. Movement of Relocated White Sharks

**Figure 3:**
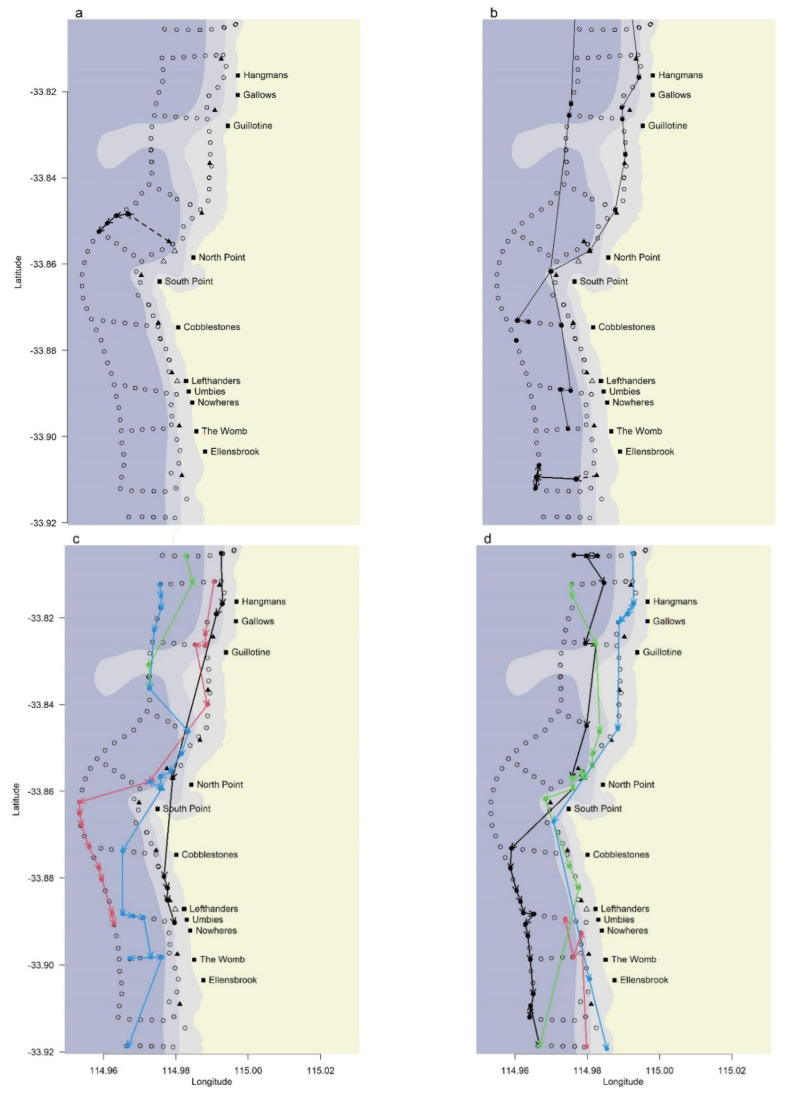
Location of VR2 (open dots) and VR4 (open triangles) receivers off Gracetown with major surf breaks (black squares) and SMART drumline locations (filled triangles) indicated. Arrows are inferred straight-line movements for sharks between successive detection locations (solid dots). Relocation paths (dashed line) to release points and detections (solid dots) are presented for: (**a**) white shark 1; and (**b**) white shark 2. All acoustically tagged white sharks that were detected within the Gracetown array while SMART drumlines were being actively fished are presented in (**c**,**d**) with colours denoting separate movements through the array.

**Figure 4:**
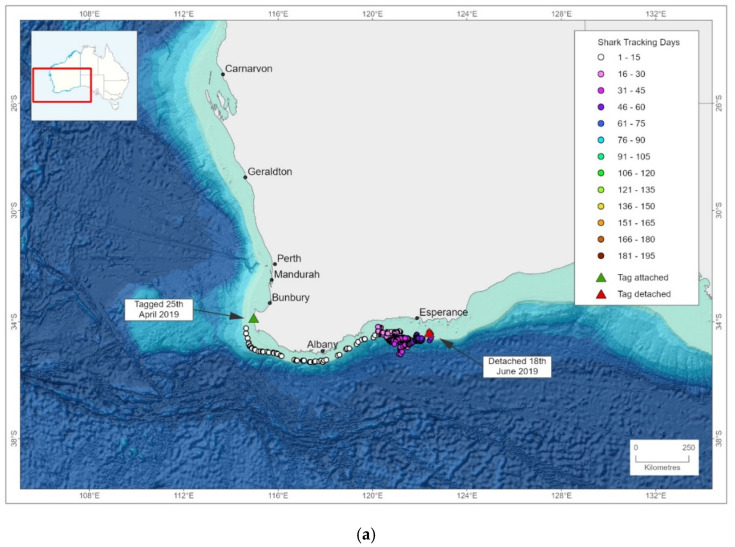

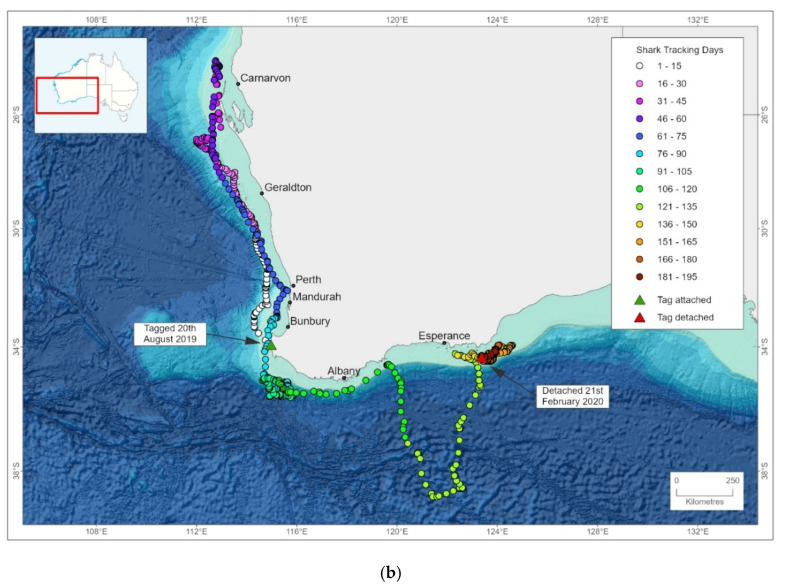
Estimated tracks of: (**a**) a 460 cm TL white shark caught on 25 April 2019, tag duration of 54 days; and (**b**) a 330 cm TL white shark caught on 20 August 2019, tag duration of 186 days. Tracks are based on model-estimated daily locations from PAT tags using GPE3.

The first white shark (460 cm TL, female) was caught on 25 April 2019 off North Point and relocated 2 km offshore. A specific relocation operation was not required due to the prevailing offshore winds, as the shark (and vessel) were already past the 1 km from shore mark at the conclusion of the tagging operations. Once released, it was detected by three acoustic receivers on the offshore line moving offshore in a southerly direction (Figure 3a). The estimated track shows that in the first 24-h the shark continued to move offshore in a southerly direction from the release site, rounded Cape Leeuwin, moved further east and arrived in waters off Esperance in May 2019 before the PAT tag released on the pre-programmed date of 18 June 2019 (Figure 4a). This shark travelled approximately 1304 km in the 54 days that the PAT tag was attached and was also subsequently recorded by the VR4 receiver at West Beach (Esperance) 235 days (16 December 2019) after its release from the SMART drumline.

The second white shark (330 cm TL, male) was caught on 20 August 2019 south of Ellensbrook. It was relocated 1 km from shore and swam directly offshore being detected on three receivers on the offshore line (Figure 3b). PAT tag tracking revealed that it moved north-west to more offshore waters and then northwards along shelf edge waters to an area west of the Houtman Abrolhos Islands in early September 2019 (Figure 4b). It continued travelling along shelf edge waters to an area north of Bernier Island before beginning a return journey southward in early October. It was detected by acoustic receivers off Perth, and 76 days after release (5–6 November 2019) it was recorded to be moving in a southerly direction through secondary arrays and subsequently the primary array at Gracetown where it was detected on the nearshore line of receivers (Figure 3b). The SMART drumlines were not being fished at the time of this latter series of detections due to risk weather. The shark then moved south-west and progressed through the Gracetown array continuing to deeper, more offshore waters in the vicinity of the Leeuwin and D’Entrecasteaux Canyons (Figure 4b). It then travelled eastward along the shelf edge waters before an extensive move southward into oceanic waters down to 38° S before heading north towards the coast in the vicinity of the Recherche Archipelago. The tag released from the animal on the pre-programmed date of 21 February 2020, 38 km from the shore in the Recherche Archipelago (Figure 4b). It travelled an overall distance of approximately 5156 km in the 186 days that the PAT tag was attached.

The second white shark was also subsequently recorded by the VR4 receiver at Frenchman Bay (Albany, Figure 1) on 16 March 2020. Detections of this white shark occurred again in the Capes region, where it was detected by 3 receivers off Injidup on 19 April 2020, before being detected again by 2 of these receivers on 22 June 2020. From Injidup it moved south through the Gracetown array being detected predominantly on the offshore line (Figure 3b). Further detections occurred in the Walpole region on 13 July 2020 and 14 July 2020, followed by detections in the north at Ningaloo on 28 August 2020. This shark was then detected in the Gracetown array on 27 October 2020 and 28 October 2020, passed through the Ocean Tracking Network line off Perth three days later and was detected in Ningaloo again on 8 November 2020. Its last confirmed detection (at the time of publishing) occurred on an acoustic receiver in the Gracetown array on 12 December 2021, 845 days after its capture on the SMART drumline.

### 3.6. Movement of other White Sharks Detected in the Study Area

Twenty-four other white sharks were detected in the Gracetown array over the first 24-months of the trial, resulting in 46 shark movements through the trial area. These included white sharks tagged as part of DPIRDs Targeted White Shark Tagging Program (*n* = 16), in addition to sharks originally tagged in South Australia (*n* = 7) and NSW (*n* = 1). Most sharks (*n* = 17) were only detected making a single shark movement, although one individual made eight separate movements through the Gracetown array. There were eight separate movements of white sharks through the array when SMART drumlines were actively being fished, which did not result in their capture (Figure 3d). 

## 4. Discussion

This study trialed the use of SMART drumlines in response to several serious incidents involving white sharks in the Capes region, WA. Of particular interest was whether white sharks, caught and relocated approximately 1 km offshore, remained offshore, which was the desired outcome from a risk-management perspective. The low catch of white sharks (*n* = 2) was insufficient to determine whether the capture, relocation and release of white sharks was an effective means of shark hazard mitigation. Nevertheless, the two white sharks captured, tagged, and relocated as part of this trial remained offshore for extended periods, reducing risks to ocean users. Whether this is a common feature of white shark capture and relocation efforts could not be determined. The study did however provide evidence to suggest that SMART drumlines pose a minimal risk to other marine species when procedures are in place to ensure rapid response times and careful handling of all animals caught. An unexpected outcome was the detection of 24 other tagged white sharks within the acoustic array that were not captured. Based on this, it is entirely plausible that other non-tagged white sharks also swam through the study region. This illustrates that white sharks are not always amenable to capture on drumlines. Our results reinforce the importance of trialing shark hazard mitigation measures under local conditions to manage human-shark interactions. 

### 4.1. White Shark Movement and Catches

The initial movements of the two captured white sharks were directly offshore after relocation and release. The first white shark (460 cm TL) was not detected again in the study region and the second shark (330 cm TL) was next detected in the Gracetown array 76 days after its capture (Figure 3b). The absence of these sharks in the study region shortly after capture is consistent with the recorded movements of white sharks caught on SMART drumlines in NSW [19]. Similarly, in Recife, Brazil, the relocation of tagged tiger sharks resulted in sharks moving to deep, oceanic waters within the first few days at liberty, without returning to the capture location [15,34]. The direct offshore movement pattern exhibited immediately after release by the two SMART drumline caught white sharks was not generally exhibited by the other previously tagged white sharks detected within the Gracetown array (Figure 3c,d). These other sharks, as well as the second white shark tagged in this study, demonstrated alongshore movements in contrast to the marked offshore movement exhibited by the two SDL relocated white sharks. 

Both white sharks caught in this study spent most of their time in offshore waters, concurring with other research on white sharks in the southern-western population [35]. The data derived from the PAT tags revealed that they detached on the dates specified for release and there was no mortality of these sharks. The PAT tag data for each white shark revealed large-scale movements that were not unidirectional, consistent with previous acoustic tracking results off WA [25]. Movement occurred as far north as Bernier Island, with both sharks moving around the south coast of WA before the tags released.

Given that the sample size for white sharks (*n* = 2) was too low to detect even a large effect (Figure 2), it was not possible to statistically determine whether SMART drumlines were effective in reducing the level of risk to ocean users in the Capes region. The Chief Scientist for WA thus made the decision to cease the trial, consistent with the outcomes of several short-term SMART drumline trials in NSW, where low catches of target species were recorded (Table 2). The careful consideration of the cost and benefits of shark hazard mitigation measures is analogous to the use of stopping rules in clinical trials based on safety, benefit, futility and cost concerns [36], and in catch and release research where fish welfare has been prioritised over statistical rigor [37]. The power analysis approach outlined here helped guide decisions regarding the feasibility of the trial, the results of which demonstrated that the sample size of white sharks was too low, despite efforts to maximize catches without compromising on animal welfare. We suggest that a similar power analysis approach could be easily adopted in other related shark hazard mitigation studies.

The decision to maintain consistency in fishing operations between this trial and those conducted in NSW was designed to provide a direct comparison between study regions (Table 2). This includes the number of hooks used, and the choice of bait (Australian salmon or sea mullet), which has successfully been used to catch white sharks in DPIRD tagging programs and the NSW drumline trials [19], and has been recorded in the stomach contents of white sharks off NSW [38]. The white shark catch rate in the current study was lower than that obtained from most SMART drumline trails in NSW (Table 2). Two seasonal white shark nursery areas have been reported for the eastern Australian population, at Corner Inlet/Ninety Mile Beach (38.854° S, 146.583° E), Victoria, and Port Stephens (32.675° S, 152.202° E), NSW [19,39]. Recent acoustic and satellite tagging of white sharks suggests that the Port Stephens nursery area extends over a 160-km stretch of coast between Forster (32.18° S, 152.51° W) and south of Terrigal (33.44° S, 151.44° W) [40]. This may explain the higher drumline catch rate at Forster in comparison to the current WA study (Table 2). Differences in catches rates between WA and NSW may also be attributed to the different marine environments and the separate populations of white sharks that occur off the western and eastern coast of Australia [21,22]. However, the large variability in catch rates of white sharks caught on SMART drumlines at different locations in NSW (Table 2) illustrates that the performance of a shark hazard mitigation measure can vary depending on local conditions. The capture of large sharks in the current study and the general lack of straightened hooks or damaged snoods indicate that the equipment used was suitable for targeting white sharks of any size. Indeed, the catch rate of tiger sharks up to 430 cm TL in the current study was considerably higher than in other regions where SMART drumlines have been used (Table 2). Although the number of false alarms in the current study was higher than that reported elsewhere [16], inspection of the fishing gear and bait remaining on the hook suggests that most of these activations were triggered by unfavourable environmental conditions or relatively small fish rather than non-hooking of the target species.

The low capture success of white sharks, as confirmed by the movement of other tagged white sharks through the Gracetown array while baited hooks were in the water (Figure 3c,d) highlights that logistical constraints and environmental conditions can influence the benefits that a specific mitigation measure may provide [41]. Other recent studies have illustrated this, with tagged bull sharks being detected in the proximity of baited hooks off Réunion Island but not taking the baits [42]. The scavenging of baits by non-target species, which included silver travelly (*Pseudocaranx georgianus*) and pink snapper in this study, can also compromise the effectiveness of fishing operations. In contrast to the low target catch in this study, DPIRD’s targeted white shark tagging program yielded a much higher capture rate across the same timeframe. Between February 2019 and May 2021, 46 white sharks were caught from 143 fishing days using similar fishing gear. Unlike the SMART drumline trial, targeted tagging is optimised around known white shark attractants that include whale carcasses and fish aggregations. This continued targeted approach results in high numbers of tagged white sharks to enable greater detection coverage in the Shark Monitoring Network and a better informed examination of white shark movements within the southern-western population.

### 4.2. Tiger Shark Catches 

Globally, the tiger shark is considered to be one of the most dangerous species of shark [43] and is a target species in other SMART drumline programs (Table 2). However, this species has not been implicated in recent shark bite incidents in the Capes region and for this reason it was not considered a target species in this trial. The catch rate of tiger sharks in this study was considerably higher than in those other SMART drumline progams (Table 2) and concurs with the results of a previous lethal shark drumline trial that was conducted off Perth and southwestern beaches [44]. The fact that more than half of the numerical catch comprised tiger sharks suggest that this species commonly occurs in the study region, particularly during the Austral summer (December to February). Despite having an apparently greater regional abundance (or susceptibility to drumline gear) than white sharks, tiger sharks appear to pose less threat to ocean users in the Capes region.

### 4.3. Animal Release Condition

As animals spent only a short time on the hook (<30 min in most cases), most animals were released in good condition, consistent with the use of SMART drumlines in other studies (Table 2). The survival of the two white sharks following their release was also confirmed by acoustic and satellite data with detections occurring more than two years after release. Blood samples taken from white sharks caught in the NSW SMART drumline program indicate that this capture method may be relatively low-stress if short response times are used, as was the case in the current trial [18]. In addition, the recapture of 11 animals from four non-target species suggests that when response times are minimised, the use of SMART drumlines poses minimal impacts to the health and welfare of these animals.

### 4.4. Stakeholder Engagement

A key feature of this trial was a high level of engagement with key stakeholders in the oversight of the program. Catch summaries and outcomes of the trial were made publicly available [45]. This ensured that the Government maintained transparency in the process. The use of third-party observers from external organisations was beneficial for informing and educating their members on the trial and providing feedback from the communities they represent on the design, implementation, and progress of the trial. Overall feedback from all third-party observers was very positive, indicating that on-board processes had been aligned to maximise animal welfare by striving to release animals quickly and in good condition. The daily deployment of drumlines, catches, and shark detections on the VR4 receivers were communicated to the wider community through the Sharksmart WA App (over 100,000 downloads as of October 2022) in near real-time. In addition, nine shark warning system towers were installed adjacent to the surf breaks. In the event of a tagged shark detection, a reported sighting, or the capture of an animal on a drumline, the lights, sirens and audio broadcasts were activated notifying beach users of shark activity within the vicinity. Collectively, these measures assisted ocean users in making their own informed decisions and enabled people to be quickly notified of a potential shark hazard. 

## 5. Conclusions

This SMART drumline trial was conducted in a challenging, high-energy environment on the south-west coast of WA, heavily influenced by large swells. Drumline operations worked effectively, and short response times ensured that the time animals spent on the hook was minimised. The initial movements of the two white sharks captured during the SMART drumline trial were directly offshore after relocation and release. The type of direct offshore movement exhibited by the two SMART drumline caught white sharks provides some evidence of an immediate reduction in risk posed by each individual shark in each instance. However, it is important to note that the sample size is insufficient to compare the movement of SMART drumline caught white sharks with other white sharks tagged outside of the trial. The implementation of the trial benefited from stakeholder engagement and the results provide further evidence to suggest that desirable animal welfare outcomes can be achieved using SMART drumlines.

## Figures and Tables

**Table 1:** Number caught (n), mean total length (TL, in cm) and sex ratio comparisons for the 352 fish caught during the SMART drumline trial. For the smooth stingray, TL = disk width, in cm. The number of males and females does not equal n for all species as it was not possible to ascertain the sex for all individuals.

Category	Species Name	Scientific Name	*n*	TL (95% CI)		Sex Ratio		
					M	F	*Χ* ^2^	*p*
Target	White shark	*Carcharodon carcharias*	2	395 (267–522)	1	1	-	-
Shark	Tiger shark	*Galeocerdo cuvier*	192	277 (268–285)	71	117	11.3	0.0008
	Bronze whaler	*Carcharhinus brachyurus*	53	262 (255–268)	46	7	28.7	<0.0001
	Shortfin mako	*Isurus oxyrinchus*	45	228 (211–245)	16	19	0.26	0.61
	Dusky shark	*Carcharhinus obscurus*	13	187 (140–233)	5	8	-	-
	Smooth hammerhead	*Sphyrna zygaena*	2	222 (91–352)	0	2	-	-
	Scalloped hammerhead	*Sphyrna lewini*	1	330 (-)	0	1	-	-
Ray	Smooth stingray	*Dasyatis brevicaudata*	37	125 (268–285)	11	20	2.61	0.11
Finfish	Pink snapper	*Chrysophrys auratus*	4	79 (64–93)	1	3	-	-
	Samsonfish	*Seriola hippos*	3	151 (145–158)	n/a	n/a	-	-

**Table 2:** Comparison of fishing gear, fishing days, target species and catch composition from SMART drumline programs. Data for NSW relates to results across all trials from 8 December 2016 to 1 December 2019, accessed on 1 December 2021 from https://www.sharksmart.nsw.gov.au/__data/assets/pdf_file/0020/1237016/sms-factsheet-smart-drumlines.pdf. Data for Réunion Island obtained from [17], and from David Guyomard (pers. com), including fishing activity from 2014 to the end of 2019; W = White shark; T = Tiger shark; B = bull shark. Unit for catch rate is 100 drum·day^−1^.

Country	State	Region	No. Drum Lines	Fishing Days	Target sp.	W Catch	T Catch	B Catch	W Catch Rate	T CatchRate	B Catch Rate	No. Fish Caught	% Alive
Australia	WA	Capes	10	539	W	2	192	0	0.04	3.56	0.00	352	98.9
	NSW	Ballina	20	695	W, T, B	136	27	9	0.98	0.19	0.06	248	99.2
		Evans Head	15	776	W, T, B	166	15	2	1.43	0.13	0.02	224	99.5
		Coffs Harbour	10	131	W, T, B	16	18	0	1.22	1.37	0.00	52	100
		Forster	10	161	W, T, B	65	2	0	4.04	0.12	0.00	84	100
		Newcastle	10	151	W, T, B	7	1	0	0.46	0.07	0.00	17	94.1
		Palm to Newport	10	169	W, T, B	3	1	0	0.18	0.06	0.00	18	88.9
		Dee Why to Manly	10	165	W, T, B	2	0	2	0.12	0.18	0.00	14	100
		Kiama	10	155	W, T, B	1	9	0	0.06	0.58	0.00	22	100
		Ulladulla	10	165	W, T, B	3	2	0	0.18	0.12	0.00	21	100
		Tathra	10	57	W, T, B	0	2	0	0.00	0.34	0.00	11	100
		Merimbula	10	58	W, T, B	0	2	0	0.00	0.34	0.00	5	100
Réunion	-	-	≤20	1410	B, T	1	160	64	0.00	0.30	0.17	429	87.0

## Data Availability

Data presented in this study are available upon request from the corresponding author.
